# Study of *Sylvilagus *rabbit TRIM5α species-specific domain: how ancient endoviruses could have shaped the antiviral repertoire in Lagomorpha

**DOI:** 10.1186/1471-2148-11-294

**Published:** 2011-10-08

**Authors:** Ana Lemos de Matos, Wessel van der Loo, Helena Areal, Dennis K Lanning, Pedro J Esteves

**Affiliations:** 1 Centro de Investigação em Biodiversidade e Recursos Genéticos, Campus Agrário de Vairão, 4485-661 Vairão, Portugal; 2 Departamento de Zoologia e Antropologia, Faculdade de Ciências, Universidade do Porto, 4169-007 Porto, Portugal; 3 Department of Microbiology and Immunology, Stritch School of Medicine, Loyola University Chicago, Maywood, IL 60153, USA; 4 Centro de Investigação em Tecnologias da Saúde, IPSN, CESPU, 4585-116 Gandra, Portugal

## Abstract

**Background:**

Since the first report of the antiretroviral restriction factor TRIM5α in primates, several orthologs in other mammals have been described. Recent studies suggest that leporid retroviruses like RELIK, the first reported endogenous lentivirus ever, may have imposed positive selection in *TRIM5α *orthologs of the European rabbit and European brown hare. Considering that RELIK must already have been present in a common ancestor of the leporid genera *Lepus*, *Sylvilagus *and *Oryctolagus*, we extended the study of evolutionary patterns of TRIM5α to other members of the Leporidae family, particularly to the genus *Sylvilagus*. Therefore, we obtained the *TRIM5α *nucleotide sequences of additional subspecies and species of the three leporid genera. We also compared lagomorph TRIM5α deduced protein sequences and established *TRIM5α *gene and TRIM5α protein phylogenies.

**Results:**

The deduced protein sequence of Iberian hare TRIM5α was 89% identical to European rabbit TRIM5α, although high divergence was observed at the PRYSPRY v1 region between rabbit and the identified alleles from this hare species (allele 1: 50% divergence; allele 2: 53% divergence). A high identity was expected between the *Sylvilagus *and *Oryctolagus *TRIM5α proteins and, in fact, the *Sylvilagus *TRIM5α was 91% identical to the *Oryctolagus *protein. Nevertheless, the PRYSPRY v1 region was only 50% similar between these genera. Selection analysis of Lagomorpha TRIM5α proteins identified 25 positively-selected codons, 11 of which are located in the PRYSPRY v1 region, responsible for species specific differences in viral capsid recognition.

**Conclusions:**

By extending Lagomorpha TRIM5α studies to an additional genus known to bear RELIK, we verified that the divergent species-specific pattern observed between the *Oryctolagus *and *Lepus *PRYSPRY-domains is also present in *Sylvilagus *TRIM5α. This work is one of the first known studies that compare the evolution of the antiretroviral restriction factor TRIM5α in different mammalian groups, Lagomorpha and Primates.

## Background

Retroviruses are RNA viruses that, when infecting a host cell, produce a viral reverse transcriptase and a viral integrase that make a DNA copy of the viral genome and integrate it into the host genome, respectively [1]. The family Retroviridae comprises a diverse range of animal viruses, including the viral genus *Lentivirus*. Lentiviruses have been isolated from primates, domestic and wild felids, and a variety of domestic ungulates (goat, sheep, cattle and horse) [2]. Until recently, all known lentiviruses were classified as exogenous (transmitted horizontally from host to host) [3]. However, in 2007, Katzourakis and colleagues [4] reported the first endogenous lentivirus identified in any species, the rabbit endogenous lentivirus type K (RELIK), present in the genome of the European rabbit (*Oryctolagus cuniculus*). RELIK has subsequently been reported in other leporid genera (*Lepus*, *Sylvilagus *and *Bunolagus*), establishing it as at least 12 million years (My) old [5,6]. These striking observations demonstrate that lentiviruses are more widespread than previously thought, extending the host range to a different mammalian order, and demonstrate that lentiviruses can be endogenized [4-6].

The intense study of lentiviruses in the past 30 years, especially of human immunodeficiency viruses (HIV-1 and HIV-2), has been more recently accompanied by the study of antiretroviral restriction factors, like the TRIM5α protein, one of the members of TRIM family [7-11]. TRIM proteins contain three domains, which together constitute the canonical TRIpartite Motif, including an N-terminal RING domain, one or two B-Box domains and a long Coiled-Coil (CC) domain [9-11]. TRIM5, like most TRIM proteins, also contains a C-terminal PRYSPRY domain, composed of four "variable loops" [9-11]. TRIM5α is the largest isoform encoded by the *TRIM5 *gene and restricts infection by HIV-1 and other retroviruses, dependent on a species-specific sequence variation in the PRYSPRY domain, upon entry into the host cell cytoplasm and prior to reverse transcription [7,8]. Each TRIM5α domain plays distinct roles in its antiviral restriction activity. The RING domain has been shown to confer E3 ubiquitin ligase activity crucial for anti-HIV restriction [12,13]. The B-box 2 domain influences recognition of the viral capsid by the C-terminal PRYSPRY domain [13-15]. The CC domain plays an important role in the restriction of viral infectivity and it is required for trimerization [11,16]. Particularly for HIV-1 and N-tropic murine leukemia virus (N-MLV) retroviruses, restriction specificity has been mapped to the PRYSPRY domain for HIV-1 and N-MLV restriction specificity is determined by both the CC and PRYSPRY domains [9,17,18]. Human TRIM5α is not effective against HIV-1 but does inhibit N-MLV, while rhesus monkey TRIM5α restricts both [19-22]. However, a single amino acid change (R332P) in the human TRIM5α PRYSPRY domain causes it to behave like rhesus TRIM5α with regard to HIV-1 restriction [17,23]. The PRYSPRY domain binds to the viral capsid, and the domain sequence variation determines the restriction specificity [17,18,24-26]. Recently, evidence from several studies began elucidating the detailed mechanism of TRIM5α activity. Also, additional activities linked to viral restriction have been described, including a role in signal transduction, the promotion of innate immune signaling and recognition of the retroviral capsid lattice [27,28]. It has been suggested that direct binding of TRIM5α to the viral capsid leads to disruption of specific inter-hexamer interfaces, causing structural damage to the capsid. TRIM5α spontaneously forms a hexagonal lattice complementary to the capsid lattice, a molecular signature of retroviruses, which greatly stimulates TRIM5α lattice formation [29,30].

Evolutionary studies of primate TRIM5α revealed a high ratio of non-synonymous to synonymous changes in the PRYSPRY domain [25,31-33]. The distribution of positively selected residues is not random, but falls in a very tight cluster at the beginning of the domain in a 13 amino acid "patch", essential for retroviral restriction and responsible, in part, for the species-specific restriction activity [31]. The same domain has also undergone length variation and segmental duplications in different primate lineages [25]. However, polymorphisms found in the *TRIM5α *coding sequence for multiple individuals from two divergent lineages of Old World monkeys (rhesus macaque and sooty mangabey), indicated that specificity varies not only between different species but also within species [34]. Despite the geographic separation and the divergence time (> 8 My), both species presented a highly similar pattern of polymorphisms, which constitutes compelling evidence for long-term balancing selection at the *TRIM5 *locus [34]. Similar evidence of selection has been recently reported for the first intron of human *TRIM5 *gene, which may affect transcription factor-binding sites and *TRIM5 *transcriptional activity [35].

Although evolutionary and functional studies of TRIM5α antiretroviral restriction activity have primarily focused on the primate lineage, *TRIM5 *orthologs have been reported in other mammalian genomes, e.g. mouse, rat, cow and European rabbit [36-39]. Active TRIM5 was identified in the European rabbit and the ability to restrict the replication of multiple unrelated retroviruses was also described [38]. Besides this leporid, Fletcher and co-workers [40] reported the restriction of divergent retroviruses by European brown hare (*Lepus europaeus*) TRIM5α and also significant differences between both leporids' TRIM5α PRYSPRY domains. These authors suggested that retroviruses like RELIK may have driven the speciation of the Old World rabbit and hare *TRIM5α *orthologs. The order Lagomorpha is divided into two families, Ochotonidae and Leporidae, which diverged around 40 My ago [41-43]. Ochotonidae contains only one extant genus, *Ochotona*, while the family Leporidae includes 11 genera where, *Lepus*, *Sylvilagus *and *Oryctolagus*, the most well-studied leporid genera, diverged around 12 My ago [41,42]. It has been suggested that the global development of temperate grasslands (7 to 5 My ago) and the formation of the west Antarctic ice sheet (6.5 My ago) promoted the development of land bridges and consequent dispersal of the genus *Lepus *from North America through Asia and into Africa [41]. The New World *Sylvilagus *lineage initially remained in North America from which it more recently colonized South America [41]. The genus *Oryctolagus *is the only leporid genus native to Europe and consists of two subspecies, *O. cuniculus cuniculus *and *O. cuniculus algirus*, which diverged around 2 My ago. The subspecies *O. c. algirus *is restricted to the southwest region of the Iberian Peninsula and a few Atlantic islands, whereas *O. c. cuniculus *has essentially a man-made worldwide distribution and includes all domestic breeds [44].

As previously suggested, considering that RELIK must already have been present in a common ancestor of these leporid genera, the hypothesis that lentiviruses might have been the driving force of the leporid TRIM5α conserved antiretroviral activity can be challenged by extending the study of evolutionary patterns of *TRIM5α *to other members of the Leporidae family, particularly to the New World *Sylvilagus *lineage. Therefore, in this study we have examined the *TRIM5α *gene of additional subspecies and species of the three leporid genera: European rabbit subspecies (*Oryctolagus cuniculus algirus*), the Old World Iberian hare (*Lepus granatensis*) and European brown hare, and the New World brush rabbit (*Sylvilagus bachmani*).

## Results and Discussion

### *TRIM5α *divergence and phylogeny in Lagomorpha

**Figure 1:**
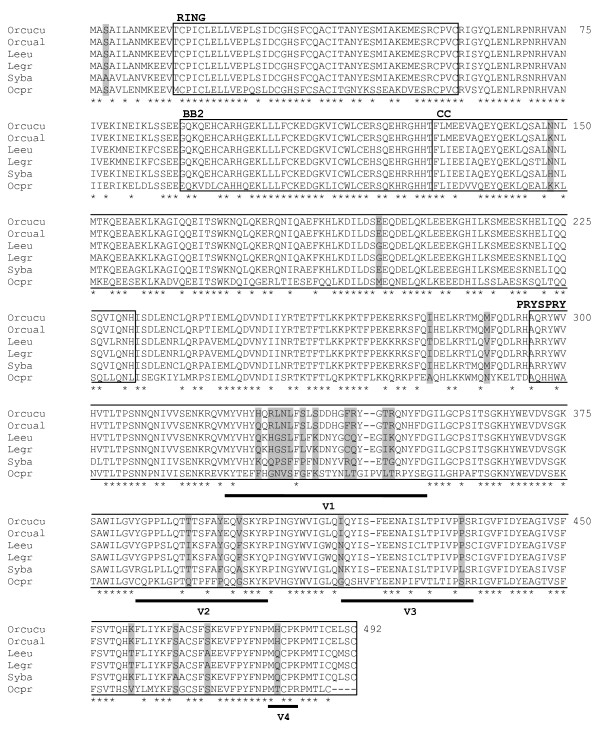
**Lagomorpha TRIM5α deduced protein sequences**. Alignment of TRIM5α deduced protein sequences from European rabbit, *Oryctolagus cuniculus cuniculus *(Orcucu) and *Oryctolagus cuniculus algirus *(Orcual) subspecies, European brown hare (Leeu - allele 1), Iberian hare (Legr - allele 1), brush rabbit (Syba) and American pika (Ocpr). Only allele 1 for Iberian hare is represented, as all the differences between both alleles are reported in the main text. European brown hare sequenced alleles were similar to those previously reported (Genbank accession numbers HM768824, HM768825) [40]. RING domain, B-box type 2 (BB2) domain, Coiled Coil (CC) domain and PRYSPRY domain, with its variable regions (v1, v2, v3 and v4), are indicated. Positively-selected codon positions are shaded; asterisk (*), identical residue between all species.

In this study, all the deduced TRIM5α protein sequences obtained from leporids were aligned and compared to that previously described for the European rabbit (subspecies *O. cuniculus cuniculus*) [38] (Figure 1). To more completely assess the *TRIM5α *gene in the Lagomorpha order, we also included American pika (*Ochotona princeps*) *TRIM5α *nucleotide sequence retrieved from the whole genome shotgun (WGS) project (contig132533, Locus AAYZ01132534). During this work, we were able to bridge the American pika protein sequence gaps previously reported [40] (Figure 1).

**Figure 2:**
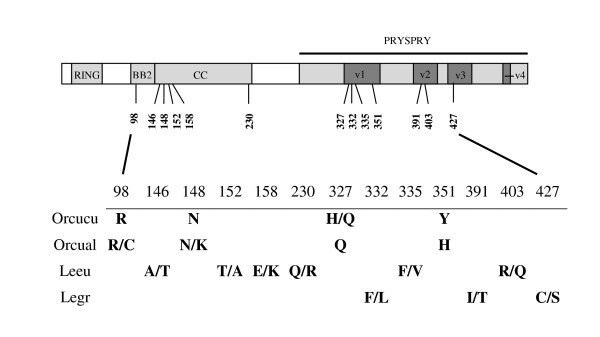
**Schematic representation of the polymorphisms within European rabbit subspecies and *Lepus *species TRIM5α**. Polymorphic sites between the European rabbit subspecies *Oryctolagus cuniculus cuniculus *(Orcucu) and *Oryctolagus cuniculus algirus *(Orcual) TRIM5α are represented. The polymorphic sites from two alleles for each *Lepus *species, European brown hare (Leeu) and Iberian hare (Legr), are also identified. Residues are numbered as in Figure 1.

The 2 My divergence between European rabbit subspecies apparently allowed the accumulation of one private residue in the *O. c. algirus *PRYSPRY v1 region (351H), different from *O. c. cuniculus *(Figure 1 and 2). Amino acid position 327 was described as being polymorphic in *O. c. cuniculus *[40], but *O. c. algirus *clones presented the same residue (327Q). In addition, two other polymorphisms were observed in *O. c. algirus *clones, one in the BB2 domain (98R/C) and the other in the CC domain (148 N/K) (GenBank accession numbers JN541226 and JN541227).

**Figure 3:**
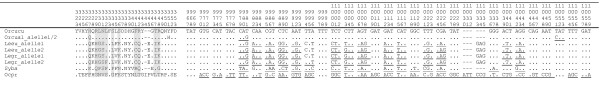
**Amino acid and nucleotide sequences of Lagomorpha PRYSPRY v1 region**. Amino acid and nucleotide sequences of PRYSPRY v1 region are represented for European rabbit subspecies *Oryctolagus cuniculus cuniculus *(Orcucu) and *Oryctolagus cuniculus algirus *(Orcual), European brown hare (Leeu - allele 1 and 2), Iberian hare (Legr - allele 1 and 2), brush rabbit (Syba) and American pika (Ocpr). The shadowed region on the amino acid representation corresponds to positively-selected sites obtained by REL analysis. Non-synonymous substitutions are underlined on the nucleotide sequences of Lagomorpha PRYSPRY v1 region. Residues are numbered as in Figure 1.

The sequencing of European brown hare *TRIM5α *confirmed the previously reported findings [40]. However, our Lagomorpha TRIM5α alignment was somewhat different from that of Fletcher and colleagues [40] due to the inclusion of new species and a more complete American pika protein sequence. Out of the 30 PRYSPRY v1 region residues, we identified 15 differing positions between the European brown hare and the European rabbit, resulting in 50% identity among v1 regions (Figure 1 and 3). *TRIM5α *from another species of hare, Iberian hare, was also sequenced in this study. With fourteen clones from two individuals, we identified two alleles differing at three positions, each of them located within the PRYSPRY variable region (332F/L, 391I/T and 427C/S) (GenBank accession numbers JN541228 and JN541229) (Figure 1 and 2). As expected, the overall identity between the TRIM5α protein sequence of European rabbit and the TRIM5α deduced protein sequence of the Iberian hare (89%) was the same as that previously reported for the European brown hare (89%) [40] which contrasted with the high divergence at the PRYSPRY v1 region (allele 1: 50% divergence; allele 2: 53% divergence) (Figure 3). The six amino acid positions previously identified as being polymorphic residues in the European brown hare [40] apparently were not polymorphic in the Iberian hare *TRIM5α *sequenced clones. In fact, all the Iberian hare clones presented the same residues as the European brown hare allele 2 in these six positions (146T, 152A, 158K, 230L, 335V and 403Q). However, four positions of all the Iberian hare *TRIM5α *sequenced clones (185K, 253D, 279I and 294R) differed from both European brown hare alleles (185E, 253Y, 279T and 294H). None of them occurred in the PRYSPRY v1 region, which was identical among *Lepus *species, except for two polymorphic positions, one in the European brown hare allele 1 (335F) and one in the Iberian hare allele 2 (332F).

The proposed leporid phylogeny estimates that the *Lepus *lineage diverged 12.80 My ago from the *Oryctolagus/Sylvilagus *clade, and that the *Sylvilagus *and *Oryctolagus *genera diverged 10 My ago [41,42]. To extend the study of leporid *TRIM5α*, we determined the *TRIM5α *nucleotide sequence from brush rabbit (GenBank accession number JN541230). As the *Sylvilagus *and *Oryctolagus *genera are closely related, a higher identity between their TRIM5α proteins was expected and, indeed, the brush rabbit TRIM5α deduced protein sequence was 91% identical to the European rabbit protein (Figure 1). However, such increase in similarity was not observed at the PRYSPRY v1 region (50% similar between these species; Figure 3). From the seven clones obtained for this species, no polymorphisms were observed.

The high divergence obtained in the PRYSPRY v1 region could be explained by gene conversion with adjacent genes. Gene conversion has been reported in other mammalian genes. For example, in leporids, a gene conversion event was observed between the two chromosomally adjacent genes *CCR2 *and *CCR5*, where the sequence motif _194_QTLKMT_199 _of the CCR5 protein was replaced by the HTIMRN motif which is characteristic of CCR2 [45,46]. In the present study, none of the chromosomally adjacent genes showed clear evidence of gene conversion with *TRIM5*, making this explanation unlikely. Furthermore, no significant BLAST matches were obtained during searches of the mammalian NCBI database. No evidence of recombination between alleles was observed.

To obtain evidence of within-species variation, the number of individuals per species should be significant. In this study, the number of individuals was low, limiting the accuracy of the observed polymorphisms. However, it cannot be ruled out that some of the sites that appeared to be variable between species are polymorphisms present within species, especially when considering the two closely related *Lepus *species. A case of *trans*-species polymorphism was reported in a study of the evolution of the immunoglobulin heavy chain variable region in *Oryctolagus *and *Lepus *[47]. In light of the previously described long-term balancing selection on primate *TRIM5α *[34,35], this scenario cannot be excluded in leporids.

**Figure 4:**
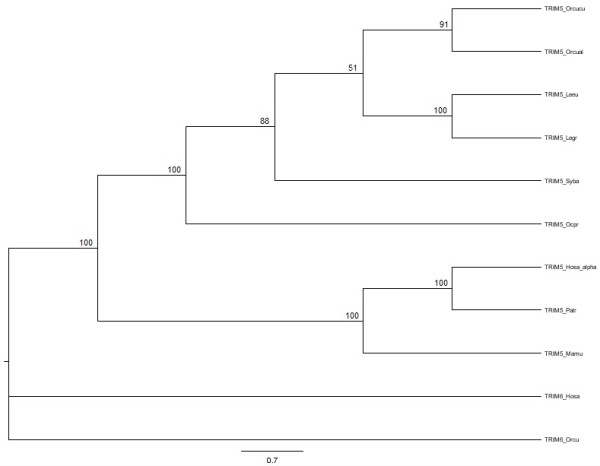
**Maximum Likelihood phylogenetic trees of lagomorph and primate TRIM5α and TRIM6 nucleotide sequences**. *TRIM5α *nucleotide sequences from Lagomorpha and three primates (human (Hosa), chimpanzee (Patr) and rhesus monkey (Mamu)), and *TRIM6 *nucleotide sequences from European rabbit and human were used to construct a Maximum Likelihood phylogenetic tree. The analyses were performed with 1,000,000 generations and 1,000 bootstrap searches. The bootstrap values are indicated on the branches.

Lagomorpha *TRIM5α *phylogenetic trees, based on nucleotide, including all described alleles, and amino acid deduced sequences, were obtained with the Maximum Likelihood method (Figure 4). *TRIM5α *nucleotide and amino acid sequences of three primates (human (*Homo sapiens*), chimpanzee (*Pan troglodytes*) and rhesus monkey (*Macaca mulatta*)), and *TRIM6 *nucleotide and amino acid sequences of European rabbit and human were also included. The trees typology was coincident with the known species tree [41,42,48,49], where *TRIM6 *sequences represented an outgroup, and primate and lagomorph *TRIM5α *formed two orthologs groups. Due to the identical typology between the two sets of data, only the tree based on nucleotide sequences is represented in Figure 4.

### Inference of positive selection in Lagomorpha TRIM5α protein

To identify a specific pattern of nucleotide substitution in the leporid TRIM5α protein, synonymous and non-synonymous substitution rates were estimated using the Nei-Gojobori method [50] and a non-synonymous to synonymous substitution ratio (d_N_/d_S_) was calculated (Table 1). Under neutrality, coding sequences are expected to present a ratio of non-synonymous substitutions (d_N_) over synonymous substitutions (d_S_) that does not significantly deviate from 1 (ω = d_N_/d_S _= 1), while significant deviations may be interpreted as either the result of positive selection (ω > > 1) or of negative selection (ω < < 1). This simple analysis showed that the ratios obtained between genera ranged from 2.8 to 4.3, clearly higher than 1, suggesting that TRIM5α is under strong positive selection.

**Table 1:** Leporid TRIM5α estimation of non-synonymous to synonymous substitution ratio (d_N_/d_S_)

	Orcucu	Orcual	Orcual	Leeu	Leeu	Legr	Legr
		allele1	allele2	allele1	allele2	allele1	allele2
Orcucu							
Orcual_allele1	1.5						
Orcual_allele2	1.5	1.0					
Leeu_allele1	3.0	3.1	3.4				
Leeu_allele2	2.9	2.9	3.2	3.0			
Legr_allele1	3.2	3.2	3.2	2.5	2.0		
Legr_allele2	3.2	3.2	3.2	3.0	3.0	n. a.	
Syba	2.8	2.9	2.9	4.1	3.8	4.3	4.1

*Oryctolagus cuniculus cuniculus *(Orcucu), *Oryctolagus cuniculus algirus *(Orcual), European brown hare (Leeu), Iberian hare (Legr) and brush rabbit (Syba).

n.a.- not applicable (no non-synonymous substitutions were observed).

The high variability of the PRYSPRY domain and the positive selection of TRIM5α described in primates [18,25,31-33] prompted Fletcher and colleagues (2010) [40] to perform a codon-based selection analysis using the random effect likelihood (REL) model. With this analysis, the authors identified 11 positively-selected codons, 4 of which were located in the PRYSPRY v1 region [40]. Using the same methodology and parameters, we identified 25 positively-selected codons, 20 of which are located in the PRYSPRY domain and, more specifically, 11 in the v1 region (Table 2). In Figure 3 the amino acid and nucleotide sequences of Lagomorpha PRYSPRY v1 region are represented and the non-synonymous substitutions are marked. It should be pointed out that the PRYSPRY domain was the site of the most intense positive selection in primates [31-33]; its v1 region was identified as the major determinant of anti-HIV-1 potency distinguishing the human and rhesus monkey TRIM5α proteins [16-18,23-25,32]. The proposed evolutionary model for primate TRIM5α in which a history of virus-host interactions led to species-specific adaptations [31] can be considered also for leporids. But again, the *trans*-species scenario between leporid species cannot be ruled out to explain the species-specific variations.

**Table 2:** Positively-selected codon positions in the Lagomorpha TRIM5α deduced protein sequences

Codon^a^	Normalized E [dN-dS]	Posterior Probability	Bayes Factor	Region	Orcu^b^	Leeu	Legr	Syba	Ocpr
3	0.30	1.00	795	-	S^0^	S^0^	S^0^	A^0^	S^0^
148	0.29	0.99	224	CC	N^0^/K^+^	N^0^	N^0^	H^0/+^	K^+^
196	0.29	0.99	179	CC	E^-^	G^0^	G^0^	E^-^	M^0^
279	0.30	1.00	1913	-	I^0^	T^0^	I^0^	I^0^	A^0^
288	0.29	0.99	178	-	M^0^	V^0^	V^0^	M^0^	N^0^
327	0.30	1.00	6611	v1	H^0/+^/Q^0^	Q^0^	Q^0^	K^+^	F^0^
329	0.30	1.00	578	v1	R^+^	H^0/+^	H^0/+^	Q^0^	G^0^
330	0.30	1.00	35572	v1	L^0^	G^0^	G^0^	P^0^	N^0^
331	0.30	0.99	405	v1	N^0^	S^0^	S^0^	S^0^	V^0^
332	0.30	1.00	33711	v1	L^0^	L^0^	L^0^	F^0^	S^0^
334	0.30	1.00	5055	v1	S^0^	L^0^	L^0^	P^0^	G^0^
336	0.30	0.99	294	v1	S^0^	K^+^	K^+^	N^0^	K^+^
341	0.30	1.00	483	v1	F^0^	C^0^	C^0^	R^+^	L^0^
342	0.30	1.00	574	v1	R^+^	Q^0^	Q^0^	Q^0^	T^0^
347	0.30	0.99	320	v1	T^0^	I^0^	I^0^	T^0^	L^0^
348	0.30	1.00	691	v1	R^+^	K^+^	K^+^	G^0^	T^0^
391	0.30	1.00	3822	v2	T^0^	I^0^	I^0^	T^0^	Q^0^
396	0.30	0.99	438	v2	Y^0^	Y^0^	Y^0^	F^0^	P^0^
399	0.30	0.99	368	v2	V^0^	F^0^	F^0^	A^0^	G^0^
415	0.28	0.98	134	v3	I^0^	N^0^	N^0^	N^0^	G^0^
434	0.30	0.99	432	v3	P^0^	P^0^	P^0^	L^0^	S^0^
457	0.29	0.99	217	-	K^+^	T^0^	T^0^	K^+^	V^0^
464	0.30	0.99	416	-	S^0^	S^0^	S^0^	A^0^	S^0^
469	0.30	0.99	418	-	S^0^	A^0^	A^0^	S^0^	S^0^
480	0.30	1.00	639	-	H^0/+^	Q^0^	Q^0^	Q^0^	T^0^

European rabbit (Orcu), European brown hare (Leeu), Iberian hare (Legr), brush rabbit (Syba) and American pika (Ocpr).

^a ^Codon positions are numbered according to the alignment in Figure 1.

^b ^Single amino acid = same for both Orcu subspecies, *Oryctolagus cuniculus cuniculus *(Orcucu) and *Oryctolagus cuniculus algirus *(Orcual); Two amino acids divided by "/" = Orcucu amino acid/Orcual amino acid

Codon physical-chemical properties are also represented: Underlined codon = non-polar amino acid; non-underlined codon = polar amino acid; **^+ ^**= positive amino acid; **^- ^**= negative amino acid; **^0 ^**= neutral amino-acid.

**Figure 5:**
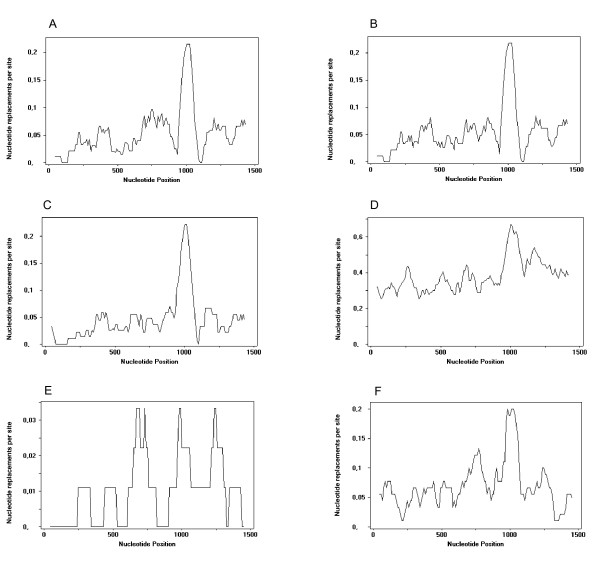
**Sliding-window analysis to detect nucleotide differences between *TRIM5α *genes from different species**. (A), (B), (C) and (D) represent the nucleotide differences between the lagomorphs European rabbit/European brown hare, European rabbit/Iberian hare, European rabbit/brush rabbit and European rabbit/American pika, respectively. (E) and (F) represent the differences in nucleotide replacements per site between the primates human/chimpanzee and human/rhesus monkey, respectively.

Our striking observation was visually reinforced by sliding-window analysis of *TRIM5α *nucleotide divergence between species (Figure 5). Comparing *Oryctolagus *with other leporid genera that diverged about 12 My ago, the nucleotide differences throughout the gene occurred primarily around 0.00-0.10 and 0.00-0.05 nucleotide replacements per site for *Lepus *and *Sylvilagus *species, respectively (Figure 5A, B and 5C). Nevertheless, the nucleotide differences peak (~0.20) was observed around nucleotide position 1000, where the PRYSPRY v1 region is located (positions 967-1059). The 40 My separation between *Ochotona *and the leporids is apparent in the 0.30 to 0.60 nucleotide replacements per site when comparing American pika to European rabbit (Figure 5D). However, the peak in the v1 region is still clearly defined. These observations can be compared to the nucleotide differences among primates. The approximately 4.5-6 My of evolutionary divergence between human and chimpanzee [48,49,51] resulted in a nucleotide difference of 0-0.03 nucleotide replacements per site, although some peaks are still defined, including one around the nucleotide position 1000 (Figure 4E). Comparison of the human and rhesus monkey also revealed a peak in the PRYSPRY v1 region (Figure 5F), variance similar to that observed among leporid genera (~0.20), although the average nucleotide differences are higher than between leporids (0.00-0.15 nucleotide replacements per site), which is consistent with the < 31 My divergence time between human and rhesus monkey [18,31].

The interest in studying ancient extinct viruses (paleoviruses) in primate genomes has increased in the past few years. However, using sequences of "modern" viruses to identify paleoviruses has been a problem and some new strategies began to be applied. The approach broadly used consists in looking for signatures of evolutionary adaptation in antiviral genes [52]. Several primate antiviral genes have already been studied and positive selection was inferred, including the focus of this paper, TRIM5α [e.g. [31,33,53,54]]. The detection of extensive diversity in primate TRIM5α led the scientific community to speculate that endogenous retroviruses and/or exogenous lentiviral pathogens may have exerted selective pressure on this host restriction factor and, in the specific case of human TRIM5α, that the acquisition of resistance to specific ancient endogenous retroviruses may be responsible for our susceptibility to HIV-1 in the present-day [31,32,55].

Assuming that selective pressure acts on the TRIM5α region that recognizes variation in the capsid of retroviruses, it was predicted that the PRYSPRY v1 region represents the interface with the capsid [18,25,31,32]. Recent studies showed that RELIK is highly similar structurally to modern-day exogenous lentiviruses and that the capacity of the capsid to form a protein-protein complex with CypA is maintained [56]. CypA is a host cell peptidyl proline isomerase that binds to the retroviral capsid [57,58]. With the absence of known exogenous lentiviruses affecting leporids, endogenous retroviruses such as RELIK were suggested to dominate leporid *TRIM5α *evolution after host germline infection [40]. Our prediction was that the TRIM5α protein from the third leporid genus known to harbor RELIK, the New World genus *Sylvilagus*, should also reflect selective pressure patterns in specific regions previously reported for the Old World *Oryctolagus *and *Lepus *genera. In fact, despite *Sylvilagus *being more closely related to *Oryctolagus*, the sequence divergence in *TRIM5α *is comparable to that found between European brown hare, Iberian hare and the two *O. cuniculus *subspecies, particularly in the PRYSPRY v1 region where the majority of positively-selected codons is concentrated.

The current release of both European rabbit and American pika genomes has increased the opportunities to identify other Lagomorpha endogenous retroviruses, remnants of ancient extinct viruses. At the same time, while comparing sequence data from several orthologs of lagomorph antiviral genes, signatures of evolutionary change in these antiviral genes could date when different ancient viral pathogens acted. Of course, it is not correct to fully assume that only one ancient retrovirus was responsible for selectively pressuring a specific antiviral gene and vice versa, or that an exogenous lentiviruses did not play a preponderant role in *TRIM5α *Lagomorpha evolution. However, until other endogenous retroviruses are identified in leporid or even lagomorph genomes and due to the absence of known exogenous lentiviruses infecting leporids, we speculate that endogenous retroviruses like RELIK could have acted as evolutionary forces on leporid *TRIM5α*.

## Conclusions

This evolutionary study on Lagomorpha *TRIM5α *gene shows a remarkable differentiation in the PRYSPRY v1 region suggesting that this gene has evolved under a high selective pressure within the Lagomorpha order. With the exception of studies on the primate lineage, this is one of the first comprehensive and detailed evolutionary studies of the antiretroviral restriction factor TRIM5α. Furthermore, the similarities observed in the species split within primates and lagomorphs allow the establishment of comparisons of the evolutionary patterns observed in *TRIM5α *gene.

## Methods

### Samples, RNA extraction and cDNA synthesis

Liver samples from one European rabbit (subspecies *Oryctolagus cuniculus algirus*; Orcual), two European brown hares (*Lepus europaeus*; Leeu) and two Iberian hares (*L. granatensis*; Legr) were supplied by CIBIO, Vairão, Portugal. In addition, two brush rabbit (*Sylvilagus bachmani*; Syba) spleen samples were provided by the Blue Oak Ranch Reserve, University of California, USA. During this study, no experimental research on animals was conducted.

Total RNA was prepared using the guanidinium thiocyanate-phenol-chloroform extraction method (TRIzol) according to manufacturer's instructions (Molecular Research Center, Inc., Cincinnati, OH, USA). First-strand cDNA was prepared from 5 μg of total RNA, using oligo(dT) primers [59] and the SuperScript™ III First-Strand Synthesis System (Invitrogen, Carlsbad, CA, USA).

### *TRIM5α *amplification, cloning and sequencing

The sequence of the European rabbit subspecies *O. c. cuniculus *(Orcucu) *TRIM5α *used in this study was taken from GenBank (accession number NM_001105673) [38]. A previously reported [40] American pika (*Ochotona princeps*; Ocpr) *TRIM5α *nucleotide sequence retrieved from the whole genome shotgun (WGS) project (contig132533, Locus AAYZ01132534) was also included.

PCR primers were designed from the available sequence for European rabbit *TRIM5α *cDNA (Forward 5'-TGTCTTGCAGAAATCTGTGAGCAAAAG-3'and Reverse 5'-AAGAGATGTACCCCAGGGTAAGAG-3'), generating an approximately 1.5 kb PCR product corresponding to the full-length CDS. The PCR thermal profile used was the following: initial denaturation (98°C for 30 s); 40 cycles of denaturation (98°C for 10 s), annealing (60°C for 30 s) and extension (72°C for 1 min); and a final extension (72°C for 10 min). Phusion^® ^High-Fidelity DNA Polymerase (Finnzymes, Espoo, Finland) was used. Finally, an additional extension step (72°C for 10 min) with Taq polymerase (GoTaq, Promega; Madison, WI, USA) was performed.

The PCR products were cloned into the pGEM-T Easy vector (Promega, Madison, WI, USA). At least seven independent clones were sequenced per allele. Sequencing was performed with an ABI PRISM 3130 Genetic Analyser (PE Applied Biosystems), following the ABI PRISM BigDye Terminator Cycle sequencing protocol. PCR products were sequenced in both directions and also with an internal primer (5' CCAACAGGAGATAACTTCCTGGAA 3'). Nucleotide sequence data obtained in this study have been submitted to GenBank and have been assigned the following accession numbers: JN541226, JN541227, JN541228, JN541229 and JN541230.

### Phylogenetic analyses

In order to infer the Lagomorpha *TRIM5α *phylogeny, based on nucleotide and deduced amino acid sequences, a Maximum Likelihood method implemented on GARLI 1.0 (Genetic Algorithm for Rapid Likelihood Inference) [60] was used. The analyses were performed with 1,000,000 generations and 1,000 bootstrap searches. The Model TIM3 +G for nucleotide substitution estimation was used as indicated by the jModelTest 0.1.1 [61]. The JTT [62] mutation model applied to the amino acid deduced sequences was used with a rate variation among sites with 4 rate categories (+G), as indicated by the program Prottest 2.4 [63-65].

### Codon-based analysis of positive selection

Most proteins appear to be under strong purifying selection most of the time, whereas positive selection is relegated to small regions of the molecule, meaning that structural and functional domains are likely to evolve at different rates [66]. A common approach to detect selective pressures involves estimating the rates of non-synonymous (d_N_) and synonymous (d_S_) substitutions [67]. The random effect likelihood (REL) model proposed by Kosakovsky and colleagues [67] and implemented in the Datamonkey web server [68] was used to identify *TRIM5α *codons under positive selection. REL involves fitting a distribution of substitution rates across sites and then inferring the rate for individual sites [67]. The REL method under the MG94xHKY85 model of evolution was used. Normalized posterior mean of the d_N_-d_S _difference and the Bayesian posterior probability for positive selection (d_N _> d_S_) for each codon position were obtained. A Bayes factor of greater than 50 suggests that a site is positively selected.

Estimation of leporid *TRIM5α *synonymous and non-synonymous substitution rates, using the Nei-Gojobori method [50], was performed on MEGA5 [69].

### Sliding-window analysis

An alternative approach to determine nucleotide substitution rate variation among different genomic regions is to plot differences as averages by sliding a window along a sequence alignment [70]. A sliding-window analysis was performed using DnaSP version 5.10 [71]. A window length of 90 nucleotides and a step size of 10 were chosen for this analysis. Nucleotide replacements per site between European rabbit/European brown hare, European rabbit/Iberian hare, European rabbit/brush rabbit, European rabbit/American pika, human/chimpanzee and human/rhesus monkey *TRIM5α *were analyzed.

## Authors' contributions

ALM participated in the design of the experiments, performed the experiments, participated in the data analyses and drafted the manuscript. HA contributed in the evolutionary analysis. DKL and WvdL participated in drafts of the manuscript. PJE conceived the study, designed the experiments and drafted the manuscript. All authors read and approved the final manuscript.
